# *MoloVol*: an easy-to-use program for analyzing cavities, volumes and surface areas of chemical structures

**DOI:** 10.1107/S1600576722004988

**Published:** 2022-06-23

**Authors:** Jasmin B. Maglic, Roy Lavendomme

**Affiliations:** a Center for Geometrical Engineering of Cellular Systems, Department of Chemistry, University of Copenhagen, Universitetsparken 5, Copenhagen, 2100, Denmark; b Center for Ordered Materials, Organometallics and Catalysis (COMOC), Department of Chemistry, Ghent University, Krijgslaan 281-S3, Ghent, 9000, Belgium; DESY, Hamburg, Germany

**Keywords:** computer programs, volume, surface area, cavities, voids

## Abstract

Cavities are a ubiquitous feature of chemical structures encountered in various fields ranging from supramolecular chemistry to molecular biology. They are involved in the encapsulation, transport and transformation of guest molecules, thus necessitating a precise and accessible tool for estimating and visualizing their size and shape. *MoloVol*, a free user-parametrizable open-source software, developed for calculating a range of geometric features for both unit-cell and isolated structures, is presented here. *MoloVol* utilizes up to two spherical probes to define cavities, surfaces and volumes. The program was optimized by combining an octree data structure with voxel-partitioned space, allowing for even high-resolution protein structure calculations on reasonable timescales. *MoloVol* comes with a user-friendly graphic interface along with a command-line interface for high-throughput calculations. It was written in C++ and is available on Windows, macOS and Linux distributions.

## Abstract 2

*MoloVol* is a free program for calculating volumes and surface areas of molecules and their cavities.

## 1. Introduction

A variety of chemical structures contain enclosed sections that are either partially or completely accessible by smaller mol­ecules. Such enclosures are known as ‘void spaces’ or ‘cavities’ and are found in a multitude of compound classes, such as enzymes, cage compounds and cavitands (Albrecht & Hahn, 2012 ▸), as well as porous solid materials (Van Der Voort *et al.*, 2019 ▸). In many cases, cavities are integral to the unique features of these compounds. Cage compounds and cavitands are, for instance, able to accommodate a single guest molecule within their cavities that can then be transformed (Fang *et al.*, 2019 ▸), transported (Zhang *et al.*, 2021 ▸) or sequestered (Lavendomme *et al.*, 2017 ▸). Porous structures, on the other hand, can additionally act as molecular sponges and accommodate large amounts of guest species (Li *et al.*, 2009 ▸). Furthermore, the effectiveness of many catalysts is tied intimately to their porosity (Sudarsanam *et al.*, 2019 ▸).

One of the most, if not the most, important criterion to determine whether one or several guests can enter a cavity is the geometrical match between the cavity and the guest molecule. There are a plethora of computer programs that aid in calculating geometrical features of cavities such as their volume or surface area: *PLATON* with its CALC VOID and CALC SOLV commands (Spek, 2009 ▸), *VOIDOO* (Kleywegt & Jones, 1994 ▸), *GRASP* (Nicholls *et al.*, 1991 ▸), *CAST* (Liang *et al.*, 1998 ▸), *HOLLOW* (Ho & Gruswitz, 2008 ▸), *Volarea* (Ribeiro *et al.*, 2013 ▸), *McVOL* (Till & Ullmann, 2010 ▸), *MDpocket* (Schmidtke *et al.*, 2011 ▸), *ProteinVolume* (Chen & Makhatadze, 2015 ▸), *3V* (Voss & Gerstein, 2010 ▸), *Voronoia* (Rother *et al.*, 2009 ▸), *PoreBlazer* (Sarkisov & Harrison, 2011 ▸; Sarkisov *et al.*, 2020 ▸), *Zeo++* (Willems *et al.*, 2012 ▸), *PyMOL* (The PyMOL Molecular Graphics System, Version 2.0, Schrödinger, LLC), *Mercury* (Macrae *et al.*, 2020 ▸), *Materials Studio* (BIOVIA, Dassault Systèmes, San Diego, USA), and the ironically named *Another Void Program* or *AVP* (Cuff & Martin, 2004 ▸). This list is not exhaustive and yet, despite this wide choice of applications, each with its own uses and perks, we could not find an application meeting the following characteristics: (i) allows for freely parametrizable calculations, (ii) exports results in an easily visualizable format, (iii) provides all desired types of volumes and surfaces (*e.g.* van der Waals, probe accessible and probe excluded surfaces), (iv) is able to analyze both unit cells and isolated molecules, and (v) operates through an easy-to-use intuitive graphic user interface (GUI).

Here we present *MoloVol*, a free user-friendly application available on all major operating systems, developed to deliver the above list of features and more. A comparative study demonstrates that *MoloVol* provides a wider range of volume and surface-area types with higher accuracy than other programs.

## 2. Accessibility and availability

*MoloVol* is an open-source application written in C++. The entire source code is available in a repository hosted on GitHub (https://github.com/molovol/MoloVol) and is free to use and modify under the MIT license. The user interface was built using the cross-platform GUI library wxWidgets, but the application is also fully functional from the command line. *MoloVol* is being actively developed and has been tested on Windows 10, macOS (10.14 and above) and Ubuntu 20.04 LTS. Additionally, we offer installation packages for Windows 7 and 8, macOS 10.11 and above, and Debian. On macOS, we provide native support for both Apple silicon and Intel processors. Current and past releases are available as precompiled binaries or as source code at https://molovol.com. Future releases will be made available through the same web page.

## 3. Overview of features

### 3.1. Method

#### 3.1.1. Volume definitions in single-probe mode

**Figure 1:**
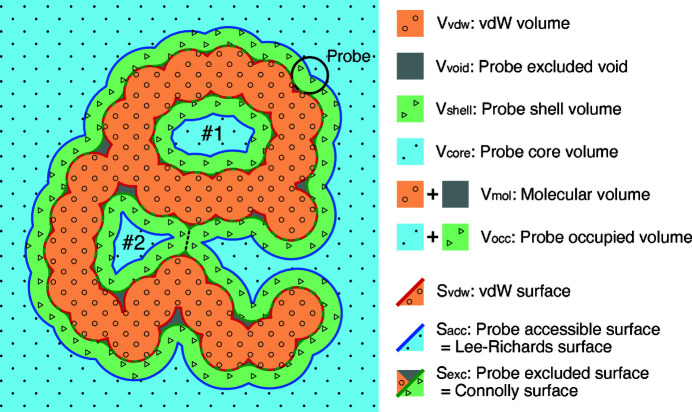
2D analog of a potential structure analysis with *MoloVol*. Types of volumes and surfaces obtained from single-probe mode. Both cavities #1 and #2 are isolated. The Connolly surface for the outside space is the molecular open surface. Dashed line: separation between cavity #2 and outside space.

*MoloVol* can determine different types of volumes that may be defined for a chemical structure. The simplest volume is the van der Waals (vdW) volume, where each atom is assumed to occupy a spherical volume (defined by its vdW radius) with its center at the atom position. The vdW volume includes any space that is occupied by atoms (orange section in Fig. 1 ▸). *MoloVol* is also able to distinguish between ‘probe accessible void’ (blue/green section in Fig. 1 ▸) and ‘probe excluded void’ volume (gray section in Fig. 1 ▸) by using a spherical probe. This ‘single-probe mode’ again assumes spherical atoms and treats them as impenetrable by the probe. Within probe accessible void volume, *MoloVol* differentiates between ‘probe core’ volume, *i.e.* sections of space reachable by the probe center (blue section in Fig. 1 ▸), and ‘probe shell’ volume, *i.e.* sections reachable by the probe but not by its center (green section in Fig. 1 ▸). Cavities are defined by spatially isolated core volume sections. For a given cavity, its total volume is the sum of the isolated core volume and all connected shell volume within one probe radius distance. However, when two cavities are close enough, they may compete for the same shell volume. In this case, the shell volume is attributed to the closest core volume, so that no shell volume is counted twice (dashed line in Fig. 1 ▸).

#### 3.1.2. Surfaces and rolling probe

In addition to volumes, *MoloVol* can be used to analyze various surfaces. The vdW surface is defined as the surface enclosing the vdW volume (red line in Fig. 1 ▸). Two more values are obtained using a spherical probe, where the probe may be imagined as ‘rolling’ over the entire structure while recording the position of the probe center and outer bound. This method was originally described by Lee and Richards (Lee & Richards, 1971 ▸; Richards, 1977 ▸) and then further developed by Connolly (1983 ▸). The ‘probe excluded surface’ is the surface traced by the probe’s outer bound and encloses the vdW volume and probe excluded void (green line in Fig. 1 ▸). The ‘probe accessible surface’ is traced by the probe’s core and additionally encloses the probe shell volume (blue line in Fig. 1 ▸). These surfaces are often referred to as ‘solvent accessible surface’ and ‘solvent excluded surface’ when the probe approximates a solvent molecule.

#### 3.1.3. Blocking open cavities in two-probe mode

A cavity is considered ‘closed’ or ‘isolated’ when a probe can neither enter nor exit, *i.e.* all entrances are smaller than the probe diameter. In proteins, isolated cavities may be considered part of the molecular volume because guest molecules do not have access to the enclosed space unless access is given through conformational motion. Using a spherical probe as described in Section 3.1.1, it is straightforward to identify isolated cavities. Open cavities that are connected to the outside such as protein tunnels, cavities of open-cage compounds and deep cavitands are, however, more difficult to analyze because there is no physical limit between the inside and outside of the molecule.

Several methods have been used to define the volumes of these open cavities, such as (i) calculating the largest sphere (Ke *et al.*, 2005 ▸; Pasquale *et al.*, 2012 ▸) or polyhedron (Liu *et al.*, 2007 ▸) that fits inside the cavity, (ii) using a larger spherical probe that cannot exit the cavity (Ronson *et al.*, 2014 ▸), (iii) arbitrarily blocking the entrances (Yamashina *et al.*, 2019 ▸), (iv) generating the convex hull of the molecule as the outside limit (Petřek *et al.*, 2006 ▸), and (v) using the probe algorithm with a probe too large to reside in the molecule to thereby define the ‘outside’ (Voss & Gerstein, 2010 ▸).

All these options involve arbitrary decisions and there is no absolute best solution. Using a large sphere or polyhedron [*i.e.* methods (i) and (ii)] artificially reduces the cavity volume by neglecting asperity within the molecule. Blocking the entrances appears the most arbitrary method and may lead to the least reproducible results if details on the blockage are not provided, but it does account for asperity. Generating a convex hull may create undesired supplementary cavities if the original structure has large concave surface regions.

**Figure 2:**
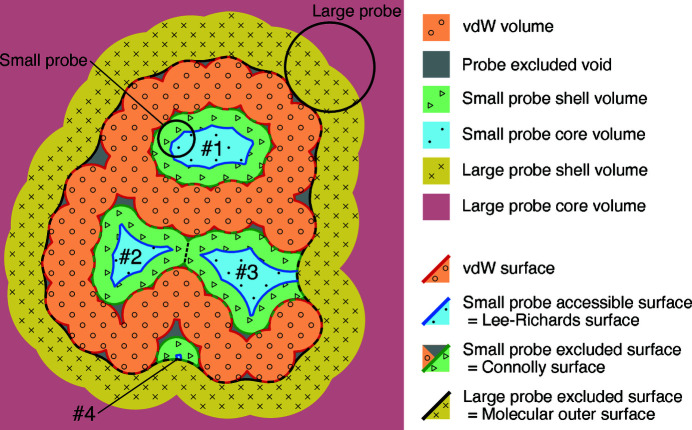
2D analog of a potential structure analysis with *MoloVol*. Types of volumes and surfaces analyzed in *MoloVol* using two-probe mode to detect internal cavities. Detected cavities are numbered. Dashed line: separation between the two touching but separate cavities #2 and #3. #3 and #4 are pockets that appear due to the large probe.

For *MoloVol*, we have opted for the fifth method using two probes: one large probe to define the outside space and one small probe to define inside cavities (see Fig. 2 ▸). In our opinion, this solution is the most elegant, as it allows defining the shape of the cavities clearly and in an easily reproducible manner using a minimal set of parameters. There are, however, two major limitations: first, in the case of very large open cavities such as for giant polyhedral structures (Fujita *et al.*, 2016 ▸), the large probe used to define the outside space must be extremely large, thereby missing the fine details of the outer shape of the molecule; second, the separation between the outside and inside space as desired by a user might not be obtainable for some intricate structures (*e.g.* in the case of protuberant spikes surrounding the entrance of cavities).

Using a single large sphere or artificial blockage to define open cavities [*i.e.* methods (ii) and (iii)] can also be realized in *MoloVol*, if preferred by the user, by changing the probe radius or loading a modified closed structure.

Owing to the translational symmetry in crystal structures, a molecule and its cavities may be translated arbitrarily within the unit cell and thereby may appear split by its edge. Thus, ‘outside’ and ‘inside’ are not as easily defined within a crystal unit cell. Nonetheless, the two-probe mode in *MoloVol* can still be of use in crystal structure analysis. For example, using two probes allows differentiation of pores of different sizes in porous materials: a large probe to explore the mesopores and a small probe to explore the micropores. The two-probe mode implemented in *MoloVol* thus has a variety of uses depending on the type of structure analyzed and the information desired. Further development for porous crystal characterization is planned (see Section 5).

### 3.2. Calculation output

**Figure 3:**
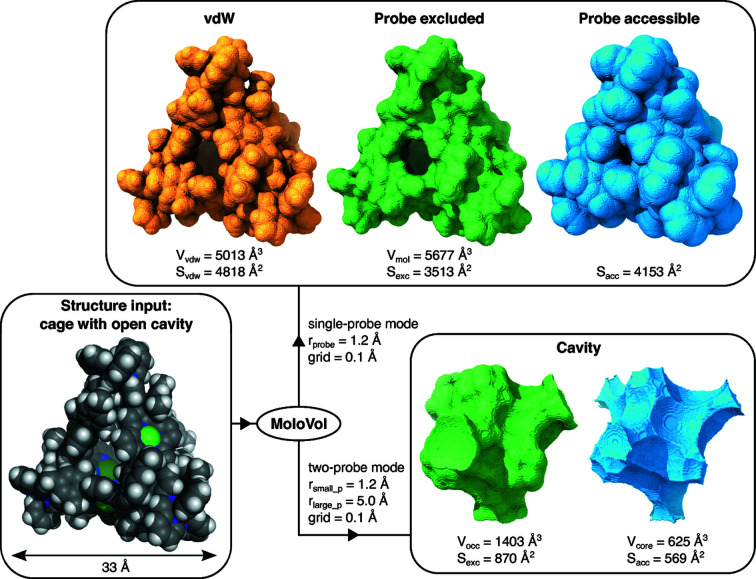
Example of the output from *MoloVol* calculations performed on an open-cage compound. The resulting surfaces were rendered in *ChimeraX* on the basis of the surface maps generated by *MoloVol*. ‘grid’ refers to the grid resolution; subscripts ‘small_p’ and ‘large_p’ correspond to the small and large probe, respectively.

As described in Section 3.1 and as seen in Figs. 1 ▸ and 2 ▸, several volumes and surfaces can be defined for a structure. This section details the types of volumes and surface areas that can be obtained from a *MoloVol* calculation. An example calculation performed on an open-cage compound (Yamashina *et al.*, 2019 ▸) with the corresponding main volumes and surface areas is shown in Fig. 3 ▸.

#### 3.2.1. Volumes

The default calculation of *MoloVol* provides the vdW (*V*_vdw_), probe excluded void (*V*_void_), probe core or accessible (*V*_core_), and probe shell (*V*_shell_) volumes. Combinations of these volumes are also calculated: molecular volume (*V*_mol_ = *V*_vdw_ + *V*_void_), probe occupied volume (*V*_occ_ = *V*_core_ + *V*_shell_) and molecular volume with isolated cavities (*V*_mol-isolated_ = *V*_mol_ + *V*_occ-isolated-cavities_), defined as the volume that cannot be accessed from the outside. Each cavity is characterized by an individual *V*_core_ and *V*_occ_ and a cavity type based on the number of entrances (only in non-unit-cell mode). In single-probe mode, *MoloVol* only differentiates between ‘Outside’ and ‘Isolated’ cavities, where the former is the probe core that reaches the boundaries of the analyzed space and the latter is every remaining cavity. In two-probe mode, the cavity type is determined from the number of entrances to the cavity from the outside defined by the large probe. It may be ‘Isolated’ for none, ‘Pocket’ for one, or ‘Tunnel’ for two or more entrances. All volume values are provided both on the molecular scale in cubic ångström and on the macroscopic scale in cubic centimetre per gram, which may be useful for comparison with experimental pore volumes in porous materials (Ongari *et al.*, 2017 ▸).

#### 3.2.2. Surface areas

In addition to volumes, surface areas can be calculated if the corresponding option is enabled. *MoloVol* provides the vdW (*S*_vdw_), probe excluded (*S*_exc_) and probe accessible (*S*_acc_) surface areas. In single-probe mode, the molecular surface area may be considered equivalent either to the total probe excluded surface (*S*_mol_ = *S*_exc_), which is redundant and therefore not explicitly given, or to the surface area that is only open to the outside (*S*_mol-open_ = *S*_exc-outside_) and encloses *V*_mol-isolated_. In two-probe mode, *S*_mol_ is defined by both probes’ excluded surfaces, but the value obtained in single-probe mode should be preferred as it better represents the asperities defined by the small probe. For each cavity, individual values for *S*_exc_ and *S*_acc_ are also given. These values are provided both on the molecular scale in square ångström and on the macroscopic scale in square metre per gram for comparison with experimental pore surface areas in porous materials (Ongari *et al.*, 2017 ▸).

#### 3.2.3. Surface maps

It is often important to visualize the surfaces belonging to the calculated volumes, to confirm by shape that the values calculated correspond to the desired volumetric objects and to share visual information. *MoloVol* does not yet provide a built-in viewer for three-dimensional models, but it is possible to export ‘surface maps’: *i.e.* volumetric maps in the OpenDX format that can be opened to visualize isosurfaces in popular and powerful rendering programs for chemistry such as *PyMOL* (The PyMOL Mol­ecular Graphics System, Version 2.0, Schrödinger, LLC), *Chimera* (Pettersen *et al.*, 2004 ▸) and *ChimeraX* (Pettersen *et al.*, 2021 ▸). A detailed guide on how to display the surfaces in these programs is provided in *MoloVol*’s user manual. The user can export both the surface map of the total structure and separate surface maps for each cavity. The map file contains all information needed to display each surface and volume type calculated by *MoloVol*.

### 3.3. Input files and parameters

#### 3.3.1. Structure files

Chemical structures can be loaded from XYZ, PDB and CIF files. XYZ files contain only a list of atoms with their symbol and Cartesian coordinates in ångström. They are often most convenient for chemical structures with no crystallographic information. Protein Data Bank (PDB) and Crystallographic Information Framework (CIF) files may contain more information, notably crystallographic information. Consequently, crystal-unit-cell analysis in *MoloVol* requires either PDB or CIF files.

#### 3.3.2. Element file

A file containing element radii and atomic weights is provided with the program binaries. Element radii were taken from a study by Alvarez (2013 ▸) because the list was more extensive than other references that we found. Atomic weights were taken from an IUPAC technical report (Meija *et al.*, 2016 ▸). These values might slightly diverge from the ones used in other programs. This file with element properties is loaded by default, but it is possible to select a different file generated by the user with other values or custom elements if needed.

#### 3.3.3. Calculation parameters

After a structure file has been imported, all atoms are listed along with their radii in a table. The radii are read from a provided element file (see Section 3.3.2). It is possible to change the radii from the default in two ways: either by changing the values in the element table directly or by loading a custom element file. Using the former method, the radii will be reset when loading a new structure. The latter method may be used if changes should apply to multiple calculations. Furthermore, loading a custom element file allows defining custom element symbols. We do not expect this function to be heavily used, but it might be useful in certain cases. Potential applications may be, for instance, setting different radii for aromatic and aliphatic carbons or for different oxidation states of the same element, or to increase the size of carbon atoms to compensate for missing hydrogen atoms in the structure as is common practice for large bio­macro­mol­ecules. Custom element symbols may only contain alphabetic characters.

The following options can be toggled easily through tick boxes in the user interface: (i) include HETATM lines in PDB files (*i.e.* atoms not belonging to the bio­mol­ecule, such as solvent), (ii) analyze the crystal unit cell, (iii) calculate surface areas (by default, only volumes are calculated), and (iv) switch between single- and two-probe mode.

The probe radii can be modified to meet the user’s needs. Common probe radii (*e.g.* hydrogen atom, water mol­ecule, argon atom) are accessible via a dropdown menu, but any value can be entered manually. Increasing the probe radii leads to an increase in calculation time (see Section 4 for the algorithmic complexity).

*MoloVol* analyzes space by partitioning it into discrete cubes or voxels (see details in Section 4). Each voxel is placed on a cubic grid. The grid resolution is defined as the voxel side length and can be changed. A smaller grid-resolution value will increase the accuracy of the calculated values but also increase the calculation time (see Section 4 for the algorithmic complexity).

Finally, a parameter named ‘optimization depth’ can be chosen. This parameter is linked to the speed of the calculation and does not influence the calculated values. This parameter does not typically need to be modified by users for common calculations but is nonetheless available to give full control of calculation parameters if desired. The effect of this parameter is detailed in Section 4.5.1.

#### 3.3.4. Graphic interface

**Figure 4:**
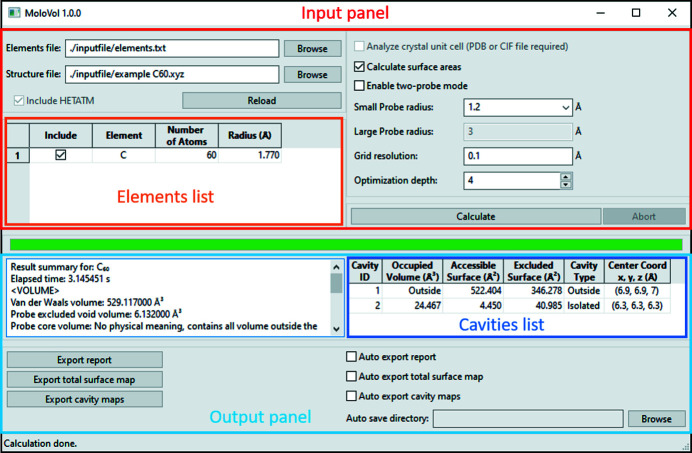
Graphic user interface of *MoloVol v1.0.0* after running a calculation. The different sections of the interface are outlined and labeled. This interface is shown as a reference but may change in upcoming versions.

*MoloVol* was designed to provide a user-friendly GUI. Its current version is shown in Fig. 4 ▸. The GUI is divided into panels that serve to spatially separate input and output. The input panel allows changing all parameters discussed above in a compact way. The element list provides the opportunity for the user to review whether all atoms were correctly imported from the structure file and allows including or excluding specific elements, as well as changing their radii before starting the calculation. The output panel contains a summary of the most important calculation results, a list of cavities sorted by decreasing volume, and a section for automatic or manual export of the complete results report and surface maps. The GUI also contains a progress bar (in green) and a status bar (at the bottom) to show the progress at different stages of the calculations.

#### 3.3.5. Command-line interface

Providing only a graphic user interface limits the throughput of calculations and makes it difficult to communicate with other applications. To avoid this, *MoloVol* provides a fully functional command-line interface (CLI), usable with the Windows command prompt or Linux/macOS terminal. This allows automating calculations and thereby increasing the throughput. A guide on how to use the CLI is provided in the user manual of *MoloVol*.

## 4. Algorithms

### 4.1. General

*MoloVol* analyzes the section of space containing a chemical structure by partitioning it in discrete sections. The space is divided into a 3D grid of small cubes (*i.e.* voxels). With respect to computational cost, this voxel approach is more scalable than analytical calculations. Moreover, once each voxel is identified, a variety of physical values, such as volume and surface types, can be derived with minimal supplementary computational time. A voxelated space also allows tuning the grid resolution (*i.e.* the side length of the voxels) to find a compromise between accuracy and calculation time. With this approach, the calculation time increases with the inverse cube of the grid resolution *g* [*i.e.* complexity *O*(*g* ^–3^)], as the number of voxels increases in each spatial dimension. However, we employ optimizations to reduce the average complexity to sub-cubic and achieve reasonable calculation times even at a high resolution (*e.g.* 0.1 Å, corresponding to 1000 voxels per Å^3^) for structures as large as proteins. For clarity, voxels are presented as pixels in subsequent figures, but the text may refer to them as voxels.

**Figure 5:**
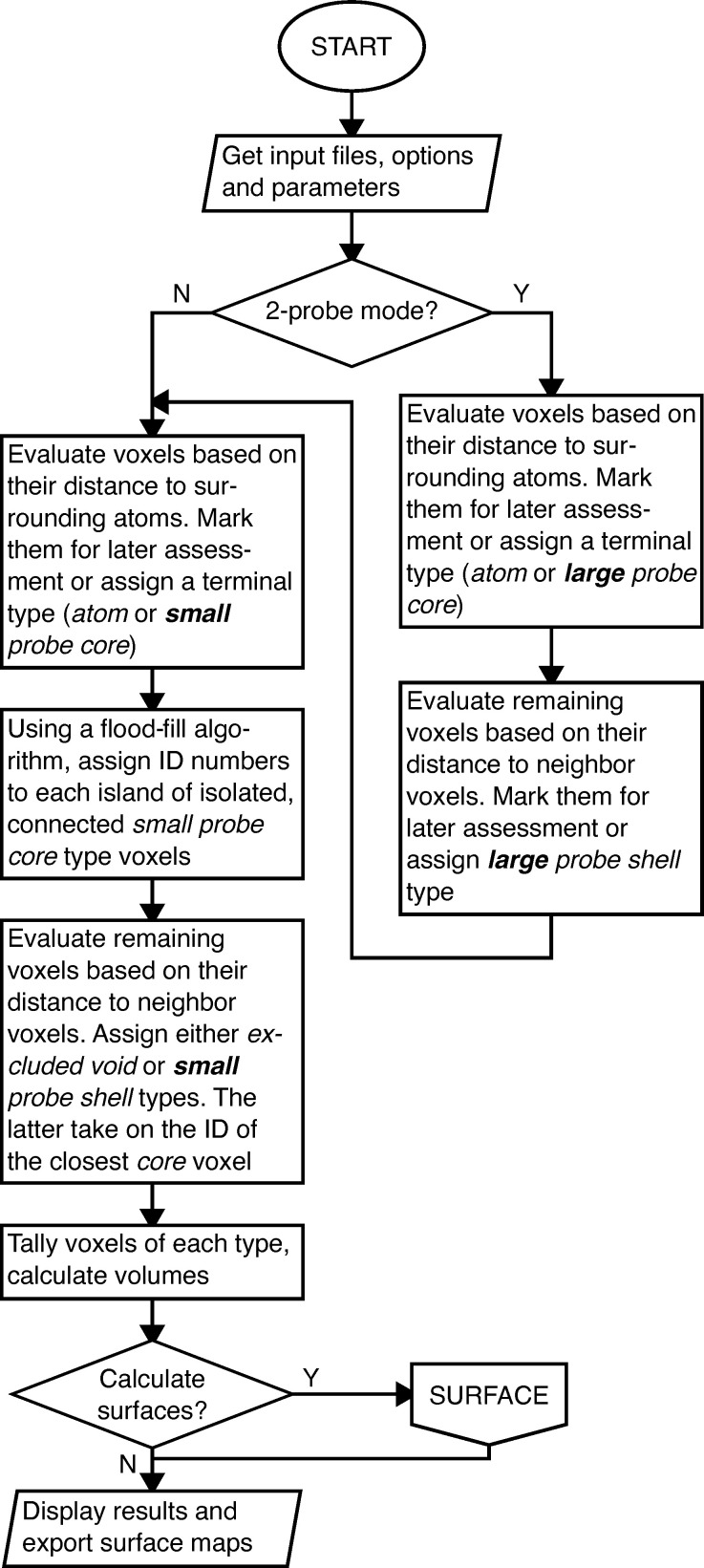
Flow chart presenting an overview of the volume calculation algorithm. Voxel types are printed in italics. The flow chart for the algorithm used for calculating surfaces is shown in Fig. S3.

A schematic overview of the program is presented in the form of a flow chart in Fig. 5 ▸. The algorithms are detailed in the following sections and additional flow charts are presented in the supporting information (Figs. S1–S3)

### 4.2. Determining voxel types

Voxels are evaluated one by one to determine whether they belong to an atom, the probe core, the probe shell or the probe excluded void volume. In single-probe mode, two subsequent algorithms evaluate voxels: the first loop determines whether a voxel is of atom type or probe core type or whether its type cannot yet be identified; the second loop evaluates the remaining unidentified voxels to determine whether a voxel is of probe shell or probe excluded void type. These algorithms are described in Sections 4.2.1 and 4.2.2. In two-probe mode, there are four loops to identify (i) atom and large probe core types, (ii) large probe shell type, (iii) small probe core type, and (iv) small probe shell and probe excluded void types. Despite the additional loops, the same two algorithms as for the single-probe mode are used.

#### 4.2.1. Atom and probe core types

**Figure 6:**
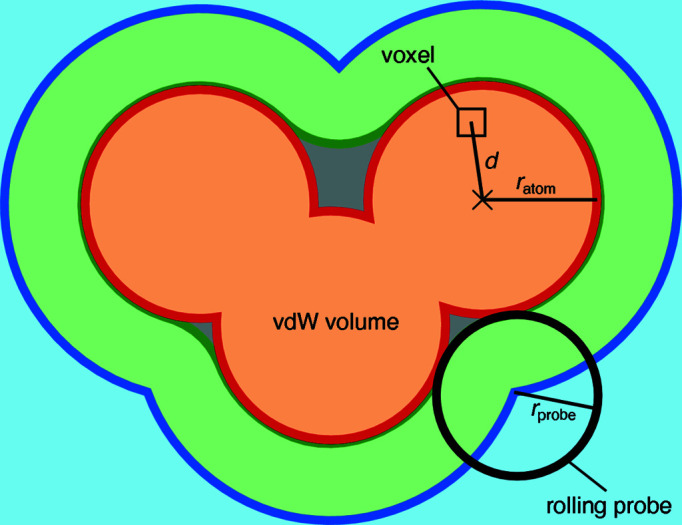
Necessary parameters and measures for determining voxel types in *MoloVol* algorithms. Voxel types: atom is orange, probe core is light blue, probe shell is light green, probe excluded void is gray.

For each voxel, its distance to each nearby atom is calculated. If, for any atom, the distance is smaller than the atom radius *r*_atom_ then the voxel is *de facto* inside an atom and set to atom type (orange in Fig. 6 ▸). Otherwise, if, for any atom, the distance *d* is smaller than *r*_atom_ + *r*_probe_ then the voxel is either of probe excluded void or probe shell type and remains undefined at this stage. If, after going through all nearby atoms, no distance matches these conditions then the voxel is assigned probe core type (light blue in Fig. 6 ▸). Nearby atoms are found using a *k*-d tree (see Section 4.5.2) to reduce the average algorithmic complexity from *O*(*mn*) to *O*(*m*log*n*), where *n* is the number of atoms and *m* is the number of voxels. A flow chart for this algorithm is shown in Fig. S1.

This is the only algorithm where the distance between voxels and atoms needs to be calculated.

#### 4.2.2. Probe shell and probe excluded void

**Figure 7:**
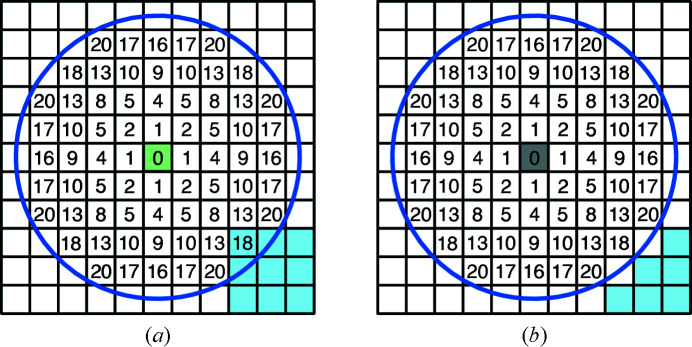
2D analog of neighbor search algorithm. Neighbor voxels are evaluated with increasing distance to the central voxel within the search radius *r*_search_ (blue circle). The square Euclidian distances between the central voxel and neighbor voxel are given in voxel units. (*a*) A core type voxel (light blue) is located within the search radius; therefore, the central voxel is assigned the shell volume type (light green). (*b*) There are no core type voxels within the search radius; therefore, the central voxel is assigned the probe excluded void type (gray).

For all voxels that remain unassigned after the first algorithm, a second algorithm determines whether the voxel is of probe shell type (light green in Fig. 6 ▸) or probe excluded void type (gray in Fig. 6 ▸) by evaluating its relationship to surrounding voxels. If the voxel has any probe core neighbor within a spherical distance *r*_search_ then it is set to probe shell type [Fig. 7 ▸(*a*)]. Otherwise, the voxel is set to probe excluded void type [Fig. 7 ▸(*b*)].

Beginning from a central voxel, whose type is yet to be determined, its neighbor voxels are evaluated in order of increasing distance. In preparation for this, the program first generates a list of relative neighbor indices sorted by the distance from the center. Absolute neighbor voxel indices are obtained through vector addition of a relative index to the center voxel index. For each unassigned voxel, the neighbors are evaluated up to the limit distance of the *r*_search_ value. The search continues until a probe core voxel is found or all relevant neighbors have been evaluated. This procedure (i) avoids calculating voxel distances, which would have a significant computational cost, (ii) ensures that only voxels in the useful range are checked, and (iii) allows the newly assigned probe shell voxel to be associated with its nearest probe core voxel, which is used for assigning shell type voxels to cavities as explained in Section 4.3. A flow chart for this algorithm is shown in Fig. S2.

**Figure 8:**
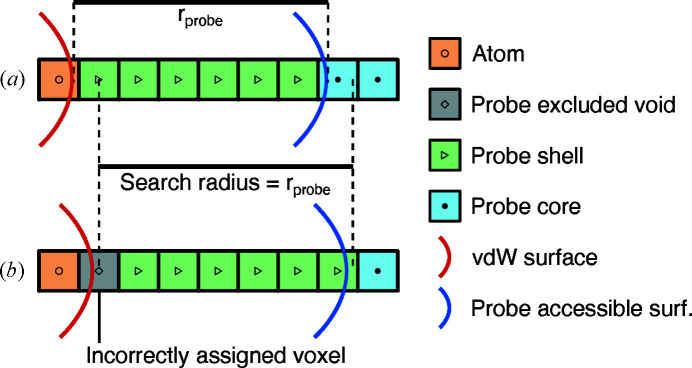
1D demonstration of how shifting the underlying voxel grid relative to the atom positions can impact the voxel types with the neighbor search algorithm. (*a*) Example of voxel types being assigned as expected when *r*_search_ is equal to *r*_probe_. (*b*) A false negative situation leading to an incorrectly assigned voxel using the same *r*_search_ and *r*_probe_ as before.

Owing to the discrete nature of the voxelated space, the distances between voxels are not continuous. This introduces spatial anisotropy with respect to the voxel grid. This is demonstrated in Fig. 7 ▸, as the furthest square Euclidian distance from the center is 16 horizontally but 18 diagonally. Another related issue stems from a propagated error in the first evaluation loop. When representing a solid in voxelated space, the voxels at the solid’s boundary will virtually always be slightly displaced. After atom and core type voxels are assigned, this displacement on both sides can lead to the gap containing unidentified voxels being significantly larger than the probe radius. Since the neighbor search algorithm checks for probe core voxels and not for the actual probe accessible surface (Fig. 8 ▸), it is necessary to increase the search radius, such that *r*_search_ = (*r*_probe_ + δ). We found that δ = 0 led to false negative checks, *i.e.* voxels that should be probe shell type were assigned probe excluded void type [Fig. 8 ▸(*b*)]. This was tested by analyzing single-atom structures in which no probe excluded void volume should be present. The ideal value of δ that eliminates false negatives entirely but does not introduce false positives depends on the grid resolution and *r*_probe_. We found no analytical value for δ. However, empirical tests with parameters expected to be used for chemical structure analysis (*i.e.* grid resolution in the range 0.05–1.0 Å and *r*_probe_ in the range 0–10 Å) have shown that δ = 2^1/2^/4, given in units of voxel side length, yielded the best results.

This part of the algorithm is typically the most time consuming in a *MoloVol* calculation. The complexity is *O*(*ms* ^3^) with *s* being the ratio between *r*_probe_ and grid resolution and *m* being the number of unidentified voxels, which itself depends on *s*. Calculations performed with a small probe radius run extremely fast at this stage and other parts become more time consuming.

### 4.3. Identifying separate cavities

One important feature of the application is to identify separate cavities and calculate their corresponding volumes and surface areas. Separate cavities are defined by portions of space between which a probe cannot travel. Thus, to identify each cavity, a flood-fill algorithm is applied to voxels with probe core type. The flood-fill algorithm evaluates all 26 neighbors to a voxel that share at least a vertex. Conducting the algorithm with only the six direct neighbors led to incorrectly isolated cavities. Indeed, the discrete voxelated space can lead to small separate islands of voxels of probe core type for cavities with sharp geometrical features that would be connected in a continuous space. This flood-fill algorithm is performed in-between the steps outlined in Sections 4.2.1 and 4.2.2. Thus, when probe shell voxels are identified by finding their nearest probe core voxel neighbor, they are directly assigned to the same cavity. Consequently, separate cavities whose shell volumes overlap are properly segmented at equidistance of their probe core regions (see cavities #2 and #3 in Fig. 2 ▸).

### 4.4. Calculating volumes and surface areas

#### 4.4.1. Volumes

Calculating volumes is trivial once voxel types and separate cavities are identified. Each type of volume is simply calculated by summing all corresponding voxels and multiplying the tally by the volume of a single voxel.

#### 4.4.2. Surface areas

Within a voxelated space, calculating surface areas is complicated by the fact that surfaces not aligned with the voxel grid will show rough steps instead of being smooth. If the surface area was calculated by simply adding the surface area of the voxels, then a voxelated sphere, for instance, would have a surface area approximating 6π*r* ^2^ instead of 4π*r* ^2^, similarly to how a pixelated circle’s perimeter approximates 8*r* instead of 2π*r*. Fortunately, algorithms have been developed to extrapolate surface-area data from voxelated objects. The marching-cube algorithm was initially developed to display smoother surfaces from a voxelated data set without consideration of the surface area and has since then been shown to be usable to extrapolate surface areas (Lindblad, 2005 ▸). Briefly, the marching-cube algorithm analyzes groups of eight voxels in a cubic arrangement. Each voxel in the octuplet is given a binary value depending on which side of the surface it is located on, and all values are stored in a byte. With respect to symmetry, there are 14 unique configurations out of the 256 possible bit combinations. Each configuration is attributed a preset surface-area parameter. Finally, the surface areas of all octuplets are summed to obtain the total surface area. A flow chart of this process is shown in Fig. S3. We implemented surface-area calculation using this marching-cube algorithm in *MoloVol* with the most recent optimal surface-area parameters reported by Lindblad (2005 ▸). The vdW surface areas calculated with *MoloVol* at a resolution of 0.1 Å were very similar to analytical values for simple systems (<0.3% error for acetyl­ene) and calculated values with other programs for large systems [1.6% difference compared with the *PyMOL* result calculated with same element radii as *MoloVol* for a cytochrome C complex containing 1356 atoms, PDB ID 6s8y (Alex *et al.*, 2019 ▸)]. Worse results were obtained with other sets of parameters for the marching-cube algorithm, notably a previous set described by Lindblad (2003 ▸). These results demonstrate that surface areas can be extrapolated from voxelated chemical structures with reasonable accuracy using a marching-cube algorithm with the right set of parameters. More details on accuracy are provided in Section 4.6.

### 4.5. Optimizations

One important aspect in *MoloVol* development was to ensure that calculations would be completed in a reasonable time even for large molecules, like proteins, and at high resolution, like 0.1 Å.

#### 4.5.1. Application of the octree data structure

Calculation time in voxel-based systems typically scales cubically with the number of voxels and thereby inverse cubically with the grid resolution. To achieve sub-cubic scaling, we adopt an octree approach. An octree is a data structure in which every node contains either eight or no children. In *MoloVol*, each voxel is an octree and may contain eight voxel children. The children have half the side length of their parents and are stacked in a 2 × 2 × 2 grid within their respective parent. For the optimization, the largest, top-level voxels are evaluated first. If their type is successfully evaluated, then the types of all descendants are also set. When the top-level voxel is a limit case, for instance by being located at the edge of an atom, then the voxel is formally divided into eight child voxels that are subsequently analyzed. The process is repeated until the lowest level of voxels is reached. At the bottom level, voxels are the smallest unit of space and are treated as zero-dimensional points, so that no limit cases can occur.

**Figure 9:**
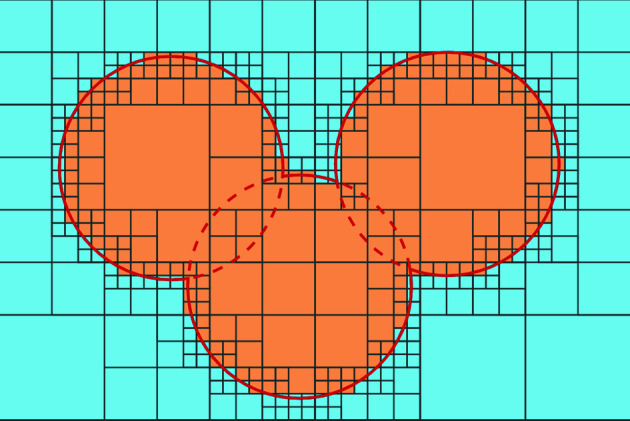
Slice of a voxelated space with octree development containing three atoms. The sizes of atoms and voxels correspond to a realistic *MoloVol* calculation with the following parameters: atom radius = 1.7 Å; probe radius = 0 Å; grid resolution = 0.2 Å; optimization depth = 3 (*i.e.* four levels in the octree including level 0).

Because of this approach, only voxels near surfaces are analyzed using smaller voxels, whereas voxels in uniform portions of the space are analyzed using larger voxels. For instance, Fig. 9 ▸ shows a 2D slice of a three-atom structure evaluated using a voxelated octree with a total of four levels. In the slice shown, we can count a total of 436 voxels (from top to bottom levels: 24, 76, 144 and 292), which is over three times smaller than the 1536 total number of voxels needed for this slice without the octree structure. The difference between these numbers is even more significant when considering the full three-dimensional space. Notably, further increasing the number of levels is counterproductive as it would, albeit slightly, increase the number of voxels to 442 for this slice with five levels. Therefore, there is an optimal value for a given calculation. Inside *MoloVol*, that value is set using the parameter ‘optimization depth’. Setting it to 0 means that no octree development occurs and no optimization is applied. Considering the typical calculation parameters and various chemical structures, we found that the optimal optimization depth is generally in the range 2–5 for different calculations. Thus, we set the default value to 4 in *MoloVol* with the possibility to change this parameter if needed. Test results are given in Section 4.6.2.

The algorithms to determine voxel types presented in Section 4.2 were adapted to be compatible with this octree optimization. Importantly, it was ensured that the results obtained from the program were invariant with respect to the optimization depth parameter. In most cases, the octree is simply exploited by conducting all computations at the highest possible octree level, only descending the tree when a limit case is identified. In the case of the flood-fill algorithm used in the cavity identification, it is necessary to evaluate neighbor voxels on varying octree levels. For this, we implemented a function that returns a list of all relevant neighbor voxels on any level.

#### 4.5.2. Optimization of atom search using a *k*-d tree

To accelerate the first algorithm in the voxel type evaluation step, a *k*-d tree was used. The *k*-d tree is a well established data structure that can be used to store a set of spatially separated points and may be used to reduce the complexity of neighbor searches (Bentley, 1975 ▸; Friedman *et al.*, 1977 ▸). Here, the *k*-d tree is used to accelerate the search of atoms that may affect the type of a given voxel.

A list of atoms is obtained from the user via an input file. Each atom has a position in 3D space, consisting of three Cartesian coordinates (*x*, *y* and *z*) and a radius. To construct the *k*-d tree, the set of all atoms is first sorted along one of the coordinates, *e.g.* *x*. The middle element of the sorted list (rounded down) is stored in the tree’s root node. Next, this procedure is repeated with the two sets of atoms left and right of the previous middle element in the sorted list; however, in the second step the atoms are sorted along the *y* coordinate. The middle elements of each list become the left and right children of the root node. This algorithm is repeated recursively, cycling through the coordinates, until all atoms have been placed in a node, resulting in a binary tree. If an atom list is empty, then the appropriate node is assigned an empty reference (NULL).

The strength of this data structure is demonstrated best with an example. In *MoloVol*, we may want to determine whether a voxel is inside any atom to determine its type. Computing the voxel–atom distance for every atom scales with *O*(*n*) with the number of atoms *n*. Using the tree, we begin by computing the distance between the voxel and the root node atom along the *x* coordinate. If the distance exceeds the largest atom radius in the tree, we can dismiss not only the node atom but one entire branch of the tree. In the best case, the comparison may reduce the number of remaining candidate atoms by a factor of 2 for each tree level, resulting in *O*(log*n*). If the distance is not larger than the largest atom radius, then the 3D voxel–atom distance must be calculated for the node atom and the search may continue along both branches.

### 4.6. Benchmarking and comparative study

Hardware specifications and input parameters are listed in detail in the supporting information.

#### 4.6.1. Effect of grid resolution on accuracy

**Figure 10:**
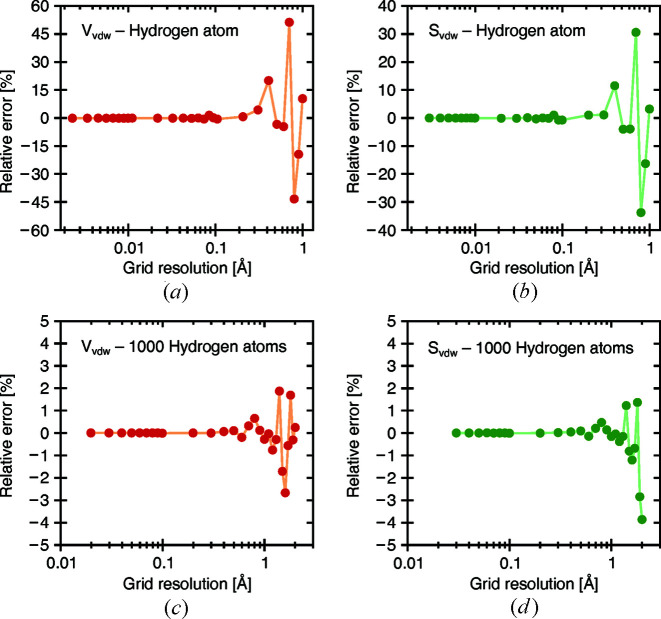
vdW volume (*a*), (*c*) and vdW surface area (*b*), (*d*) calculations at different resolutions for a single hydrogen atom (*a*), (*b*) and 1000 separate, randomly scattered hydrogen atoms (*c*), (*d*) (radius 1.2 Å). Connecting lines have been added for visual clarity. Owing to the simplicity of the structure, analytical values can be easily calculated. As the grid resolution decreases, so does the voxel size, and the results converge towards the expected values.

Calculations on a structure file containing a single hydrogen atom (radius 1.2 Å) were performed at various grid-resolution values to demonstrate the convergence behavior of the vdW volume and vdW surface-area results. Analytical values for volume and surface area can be trivially obtained because of the geometric simplicity of the single-atom structure. Fig. 10 ▸ displays the relative error versus the grid resolution for both vdW volume [Fig. 10 ▸(*a*)] and vdW surface [Fig. 10 ▸(*b*)]. As expected, the results deviate strongly and unpredictably from the analytical values at large grid-resolution values when the voxels are larger. As the grid resolution decreases, so do the voxel sizes, and the obtained results soon converge towards the expected values. We have found that good results were generally obtained at below 0.2 Å grid resolution. As such, the default grid resolution in *MoloVol* is set to 0.2 Å. An analogous test with 1000 non-overlapping hydrogen atoms shows the same trend [Figs. 10 ▸(*c*) and 10 ▸(*d*)]; however, the relative error never exceeds 3 and 4% (for volume and surface area, respectively), even at a grid resolution of 2 Å. This is because the error is stochastically compensated. Depending on the property, sufficient accuracy may therefore be achievable in larger structures using higher grid-resolution values.

**Figure 11:**
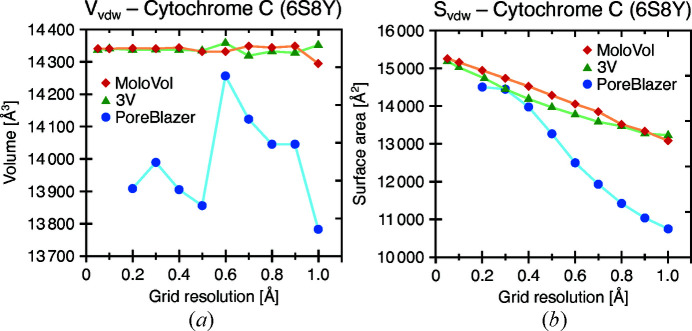
(*a*) vdW volume and (*b*) vdW surface-area calculations at different resolutions for a cytochrome C complex (PDB entry 6s8y) for different programs. Connecting lines have been added for visual clarity. *MoloVol* (red diamonds) and *3V* (green triangles) perform similarly, while *PoreBlazer* (blue spheres) deviates significantly and does not converge in the volume calculation. Smaller grid-resolution values could not be tested with *PoreBlazer* owing to the program crashing.

For a comparison of convergence behavior, the vdW volume and surface area of a cytochrome C complex (PDB entry 6s8y, 1356 atoms) were calculated at different grid-resolution values using *MoloVol*, *3V* and *PoreBlazer*. The results were plotted against the grid resolution and are shown in Fig. 11 ▸. For both volume and surface-area calculations, *MoloVol* and *3V* perform remarkably similarly, both in value and in convergence. Both show clear convergence behavior for the volume calculation and similar deviation from the mean. For the surface-area calculations, both *MoloVol* and *3V* produce a linear relationship between surface area and grid resolution, but no convergence. In analogous plots for acetyl­ene [Fig. S5(*a*)], for which analytical results are available, and for fullerene C_60_ [Fig. S5(*b*)] a similar linearity emerges, but only at small grid-resolution values. We infer that this linear trend is inherent to voxelated objects as the surface becomes more detailed at finer resolution. Structures containing a small number of atoms such as acetyl­ene and fullerene C_60_ appear to be subject to larger fluctuation at higher grid-resolution values. We hypothesize that this can be attributed to a lack of error compensation, as discussed above.

*PoreBlazer* performs differently from both *MoloVol* and *3V*. For the vdW volume, *PoreBlazer* does not reveal any convergence behavior in the studied grid-resolution regime, and the result at 0.2 Å is 3% lower than *MoloVol*’s result. For the surface area, *PoreBlazer*’s result at the finest grid resolution is in good agreement with *MoloVol*’s, but at larger grid resolution a larger disagreement is observed. In addition, *PoreBlazer*’s results follow a sigmoid curve rather than a linear trend. The performance of *PoreBlazer* is further discussed in Section 4.6.3.

#### 4.6.2. Runtime optimization due to octree development

**Figure 12:**
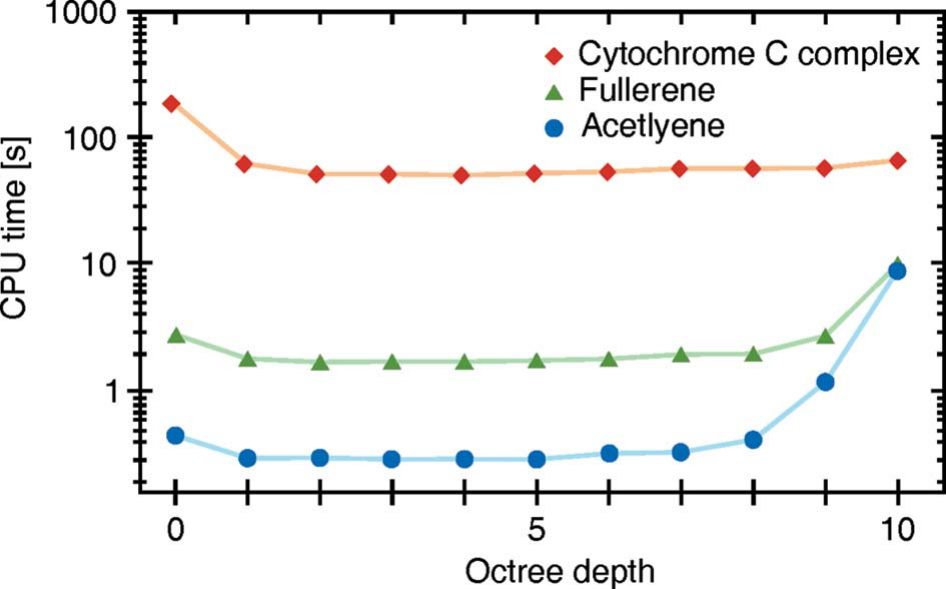
Calculation times for structures at varying octree depths. Connecting lines have been added for visual clarity. The structures were chosen to cover a wide range of atom numbers, with acetyl­ene at four (blue dots), fullerene at 60 (green triangles) and a cytochrome C complex at 1356 (red diamonds; PDB ID 6s8y; Alex *et al.*, 2019 ▸).

A range of diverse calculations were conducted to find whether an optimal value for the octree depth (see Section 4.5.1) could be determined. To test for unexpected effects of the optimization, different calculation parameters and options were varied, such as the number of atoms, addition of surface calculations, unit-cell mode and two-probe mode. Fig. 12 ▸ shows the results for three structures of different atom counts, with acetyl­ene at four, fullerene at 60 and a protein complex at 1356 (PDB entry 6s8y; Alex *et al.*, 2019 ▸). Each calculation set displays a sharp drop going from 0 to 1 octree depth, as this is equivalent to enabling the optimization. Furthermore, in all tests the optimal depth was found to be between 2 and 5. At higher depths the calculation time increases drastically as unnecessary voxels appear. On this basis, we have chosen the default octree depth to be 4.

#### 4.6.3. Calculation result comparison with other programs

Finally, we compared calculated values for diverse structures including small molecules, a protein and a porous material. Table 1 ▸ shows a summarized comparison for acetyl­ene and fullerene C_60_. The complete table and calculation details are provided in the supporting information.

Acetyl­ene is geometrically simple enough to calculate the exact volumes and surface areas analytically as references (see supporting information). *MoloVol* is the only program from those tested that provides all types of volumes and surface areas with such accuracy compared with the analytical values (<0.2% error for volumes; <1% error for surface areas).

*PyMOL* only provides means to calculate *S*_vdw_ and *S*_acc_ as it uses a completely different calculation method only suitable for surfaces around spheres. Both values are in excellent agreement with the analytical reference. Such agreement on surface areas between *MoloVol* and *PyMOL* despite them using entirely different calculation approaches, provides a solid indication that the algorithms are sound.

Across all structures, *V*_vdw_ and *V*_acc_ calculated with *3V* are in excellent agreement with the respective values calculated with *MoloVol* (<0.3% difference) as well as with the analytical values for acetyl­ene. *V*_mol_, however, exceeds the analytical value for acetyl­ene by 6% and the values of all other structures by 2–4%, in contrast to *MoloVol*. This overestimation is consistent with results obtained from *MoloVol* when not correcting for misattributed voxels, *i.e.* at δ = 0 for the search distance as discussed in Section 4.2.2 and Fig. 8 ▸, and therefore suggests a similar underlying issue. For *S*_vdw_, *S*_exc_ and *S*_acc_, *3V* demonstrates an error of up to 3% for acetyl­ene but produces similar values to *MoloVol* for all larger structures (≤1% difference). Note that the algorithm used by *3V* for calculating surface areas (Windreich *et al.*, 2003 ▸) differs from the one used by *MoloVol*. We purposefully chose not to use this algorithm in *MoloVol* because it produces different results depending on which side of a surface the area is calculated from. In summary, *3V* and *MoloVol* perform similarly well; however, *MoloVol* seems slightly better suited for analyzing small molecules.

*PLATON*’s CALC SOLV routine calculates the probe occupied volume (*V*_occ_) in a crystalline unit cell. Subtracting this *V*_occ_ from the total volume of the unit cell *V*_unit-cell_ produces *V*_mol_. Performing the calculation with a probe radius of 0 Å should provide *V*_vdw_. However, as shown in Table 1 ▸, *PLATON* performs relatively poorly in calculating *V*_mol_ and especially *V*_vdw_ for acetyl­ene, with errors of 5 and 39%, respectively. Across all other molecules *V*_vdw_ deviates by between 20 and 30% from the values calculated by all other programs. Investigating this difference in performance falls out of the scope of this study. It should only be established that *PLATON* is not suited for calculating *V*_vdw_. Interestingly, *PLATON* performs almost exactly like *3V* for *V*_mol_ (≤0.6% difference). Therefore, the previous discussion for *3V* is equally valid for *PLATON*.

*Zeo++* uses algorithms based on Voronoi tesselation which are significantly different from the voxel-based approach in *MoloVol*. The values calculated with *Zeo++* are in good accordance with those from *MoloVol* with the exception of *V*_mol_, which appears to be systematically larger.

*PoreBlazer* tends to underestimate *V*_vdw_ and *V*_mol_ for all structures, while it overestimates *S*_acc_ for small molecules and underestimates *S*_acc_ for larger structures. This discrepancy may originate from a hard-coded coefficient that increases the radii of the atoms and probe by a factor of 1.122 in some algorithms of *PoreBlazer v4.0* (Sarkisov *et al.*, 2020 ▸). According to the authors, this coefficient was introduced to more accurately reflect a monolayer of adsorbate. Values calculated with *PoreBlazer* are, therefore, not comparable with the results of other programs that aim to describe the geometrical features rather than a realistic physical system with an adsorbate. Another possible source of error comes from the use of a random sampling to calculate surface areas.

This comparative study demonstrates that *MoloVol* provides more volume and surface-area data than most other programs. However, other programs are clearly specialized for use in a certain field of chemistry, *e.g.* *3V* being devoted to biomacromolecules or *Zeo++* and *PoreBlazer* being devoted to porous materials. Accordingly, these programs provide additional features useful to their respective fields, such as the maximum pore diameter in porous material, and cannot be replaced entirely by *MoloVol* in its current version.

We stress that obtaining all results from programs other than *MoloVol* may require compiling the program from source code, modifying the source code (*e.g.* changing hard-coded element radii), using the command line, manipulating input files (notably, to create dummy unit cells around isolated molecules for programs that only analyze crystal structures) and/or deriving desired values via indirect approaches (see details in the supporting information). In contrast, *MoloVol* calculations were performed directly on the unmodified structure files in a few clicks from the GUI. We believe that this user friendliness is one of the greatest strengths of *MoloVol*, as it makes these calculations accessible to a larger user base.

#### 4.6.4. Performance comparison with other programs

**Figure 13:**
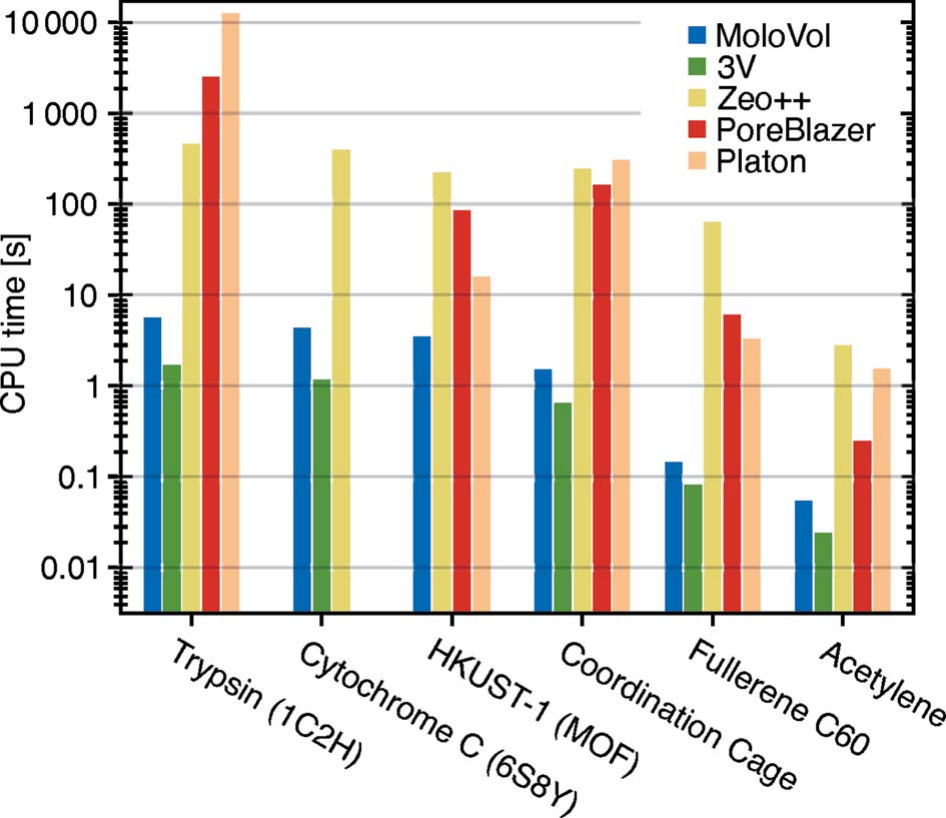
Runtime comparison between *MoloVol* and several other programs. Calculations were conducted in all applications using the same input parameters when applicable (*i.e.* atomic radii, probe radius and grid resolution). *MoloVol* performs faster than *Zeo++*, *PoreBlazer* and *PLATON* and slightly slower than *3V*.

In a comparative study, six structures were analyzed using *MoloVol* and several other programs. All programs are compared with respect to the runtime in Fig. 13 ▸. An effort was made to ensure optimal comparability, such as using the same element radii and comparable structure files. However, it is not feasible and sometimes not possible to ensure perfect comparability, as each program provides its own unique set of molecular properties and may require additional input parameters that lack an analog in *MoloVol*. The runtime was measured excluding idling.

Details on how the calculations were performed are provided in the supporting information. Briefly, a *MoloVol* calculation was performed for each structure using *MoloVol v1.0.0.* The calculations were run in single-probe mode with a probe radius of 1.2 Å, a grid resolution of 0.2 Å and an optimization depth of 4. For the other programs (*3V*, *PLATON*, *Zeo++*, *PoreBlazer*) the same calculation parameters were chosen wherever applicable. In all cases, the atomic radii were changed to *MoloVol*’s default values. Not all programs directly provide the same properties as *MoloVol*. For some programs this could be compensated for by running multiple calculations to obtain a specific value. The contributions from multiple calculations are broken down in Figs. S6–S8. Some programs (*PLATON*, *Zeo++*, *PoreBlazer*) require crystal unit cells, so dummy cells were prepared to allow for their use. Finally, *3V* was compiled without OpenMP support (parallel processing on multi-core CPU), as this is the default configuration. A knowledgeable user could compile a version with OpenMP support and obtain faster runtimes than we report.

Fig. 13 ▸ shows that across all structures *MoloVol* performed consistently faster than *Zeo++*, *PoreBlazer* and *PLATON*, often by more than a factor of 10. Overall, *3V* performs slightly but consistently faster than *MoloVol*. HKUST-1 could not be evaluated with *3V* because it does not support crystal unit cells. Despite the slightly slower calculation times when compared with *3V*, *MoloVol* provides overall more output results in a single calculation, such as cavity properties.

## 5. Limitations and planned features

*MoloVol* treats atoms as static impenetrable spheres. This is a common and convenient model but does not represent the complex reality of atomic interactions and molecular motion. As such, the values calculated with *MoloVol* can be informative and help describe and compare molecular systems but should by no means be considered absolute limits to, for instance, what guest can or cannot reside in a cavity.

In its current state, *MoloVol* can be used to identify, locate and analyze cavities within porous crystalline materials. Yet, for crystal structure analysis a user may need to manually distinguish continuous pores from isolated cavities (Ongari *et al.*, 2017 ▸). We plan to include a feature that identifies pores automatically in a future version.

*MoloVol* can currently provide a list of cavities with their volumes, surface areas, center positions and cavity types, based on the number of openings in two-probe mode. We plan for *MoloVol* to indicate connecting and neighboring cavities and present a network of cavities in the report to provide more detailed spatial information. This feature is meant to help users identify useful cavities more easily.

Considering the anisotropy of a voxelated grid, rotating or translating the input chemical structure may lead to slightly different calculated volumes and surface areas. We are considering adding a feature to randomize the structure’s orientation and provide an average result from multiple calculations. This reduces anisotropy and may increase accuracy.

Visualizing surfaces currently requires the use of third-party software. Adding the ability to visualize the calculated surfaces directly within the *MoloVol* user interface is under consideration.

Despite the algorithmic optimizations we have implemented in *MoloVol* that ensure fast calculations, there remains a clear optimization strategy that has yet to be exploited. It is possible to further accelerate calculations by parallelizing the program instructions, using either GPU- or CPU-driven multithreading. Such features are not critically needed but are nonetheless under consideration for future versions.

*MoloVol* is designed as a general tool for analyzing geometrical features of chemical structures. As such, it cannot compete with software dedicated to more specific functions, such as analyzing the chemical interactions within cavities. Yet, its simplicity of use and, to the best of our knowledge, its wider range of geometrical features that can be calculated compared with other available software makes *MoloVol* a useful tool for several fields of chemistry, including but not limited to host–guest chemistry, porous materials and structural biochemistry.

## Supplementary Material

Flow charts of algorithms, calculations comparison between different programs and computational details. DOI: 10.1107/S1600576722004988/yr5079sup1.pdf

## Figures and Tables

**Table 1:** Comparison of volumes and surface areas calculated for acetyl­ene and fullerene C_60_ with different programs All programs were run with the same input parameters where applicable (*i.e.* van der Waals radii H = 1.20 Å, C = 1.77 Å; grid resolution = 0.2 Å; probe radius = 1.2 Å). Extended tables are available in the supporting information with all calculation details.

Structure	Method	*V*_vdw_ (Å^3^)	*V*_mol_ (Å^3^)	*V*_acc_ (Å^3^)†	*S*_vdw_ (Å^2^)	*S*_exc_ (Å^2^)	S_acc_ (Å^2^)
Acetyl­ene	Analytical	37.80	37.95	153.75	57.47	56.55	141.82
	*MoloVol*	37.74	37.84	153.74	57.00	56.98	141.48
	*PyMOL*	–	–	–	57.52	–	142.05
	*3V*	37.84	40.08	153.896	55.68	58.20	139.50
	*PLATON* ‡	23	40	–	–	–	–
	*Zeo++* ‡	38.79	39.00	153.74	57.41	–	141.91
	*PoreBlazer* ‡	35.80	35.90	–	–	–	165.78

C_60_	*MoloVol*	528.62	537.66	1077.97	400.10	385.38	526.75
	*PyMOL*	–	–	–	404.28	–	529.93
	*3V*	528.90	555.50	1077.08	397.74	395.99	520.99
	*PLATON* ‡	405	555	–	–	–	–
	*Zeo++* ‡	531.43	566.91	1080.88	404.53	–	527.27
	*PoreBlazer* ‡	508.47	519.28	–	–	–	536.03

† This value has little physical meaning but is still provided by *3V* and thus included here for comparison. For other programs, it is derived from other values: *V*_acc_ = *V*_vdw_ + *V*_void_ + *V*_shell_ = *V*_unit-cell_ − *V*_core_ (for crystal structures).

‡ These programs require crystal structure input; therefore, a dummy unit cell was built around the structures.
