# Supplementary material for: MoloVol: an easy-to-use program for analyzing cavities, volumes and surface areas of chemical structures
Source: J Appl Crystallogr. 2022 Jun 23;55(Pt 4):1033–44. doi: 10.1107/S1600576722004988 (PMC9348874; doi:10.1107/S1600576722004988)
Supplement: Supplementary file 1 [file j-55-01033-sup1.pdf]

JOURNAL OF APPLIED CRYSTALLOGRAPHY

Volume 55 (2022)

Supporting information for article:

MoloVol: an easy-to-use program for analyzing cavities, volumes, and surface areas of chemical structures

Jasmin B. Maglic and Roy Lavendomme

MoloVol: an easy-to-use program for analyzing cavities, volumes, and surface areas of chemical structures

Supporting Information

Jasmin B. Maglic[1] and Roy Lavendomme[2]*

[1] Center for Geometrical Engineering of Cellular Systems, Department of Chemistry, University of Copenhagen, Universitetsparken 5, Copenhagen, 2100, Denmark

[2] Center for Ordered Materials, Organometallics and Catalysis (COMOC), Department of Chemistry, Ghent University, Krijgslaan 281-S3, Ghent, 9000, Belgium

Correspondence email: molovol@outlook.com

Table of Contents

1. Flow charts of algorithms 2
2. Analytical volume and surface area values for acetylene 5
3. Pratical aspects of computations 7
  3.1. Hardware specifications 7
  3.2. Common considerations 7
  3.3. MoloVol 7
  3.4. PyMOL 8
  3.5. 3V 8
  3.6. Platon 8
  3.7. Zeo++ 9
  3.8. PoreBlazer 9
4. Computational results 11
5. Breakdown of runtime contributions 13
6. Input structures 15
  6.1. Acetylene 15
  6.2. Fullerene C_{60} 15
  6.3. Metal-organic cage 16
  6.4. Cytochrome C complex 16
  6.5. Trypsin protein 16
  6.6. HKUST-1 16

1. Flow charts of algorithms

The general process of a MoloVol calculation is shown in Fig. 5 of the manuscript. To provide more insight into the details of the calculations, three flow charts are presented in Fig. S1-S3. These cover integral algorithms that are used during the main volume and surface calculation. These algorithms are described in more detail in the manuscript.

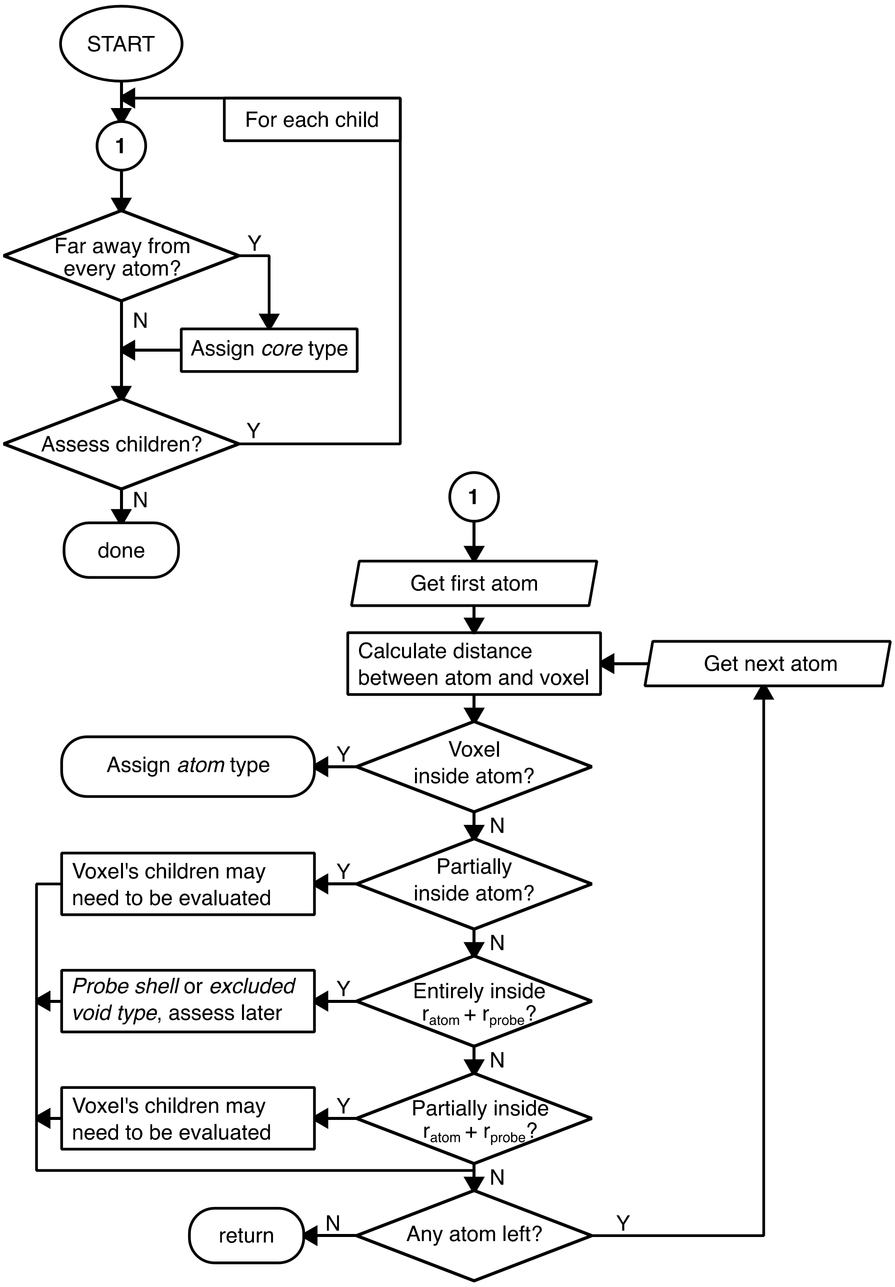

Figure S1. Flow chart depicting the algorithm that differentiates between atom and probe core type voxels. After the voxel-atom distance is evaluated in (1) and none of the comparisons have

turned out true, the voxel is considered “far away from every atom” and assigned probe core type. This algorithm is described in section 4.2.1 of the manuscript.

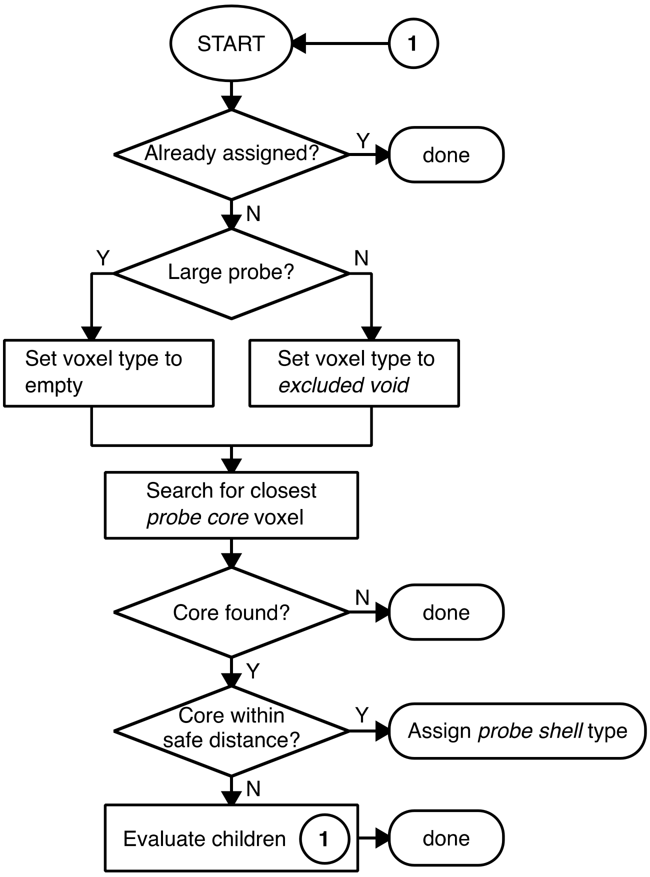

Figure S2. Flow chart depicting the algorithm that differentiates between probe excluded void and probe shell type voxels. Prior to the search for a neighboring core type voxel, the voxel is assigned a default type. The voxel will retain this type if no core voxel is found. The default type depends on whether this algorithm is conducted with the large probe (as part of two-probe mode) or with the small probe. This algorithm is described in section 4.2.2 of the manuscript.

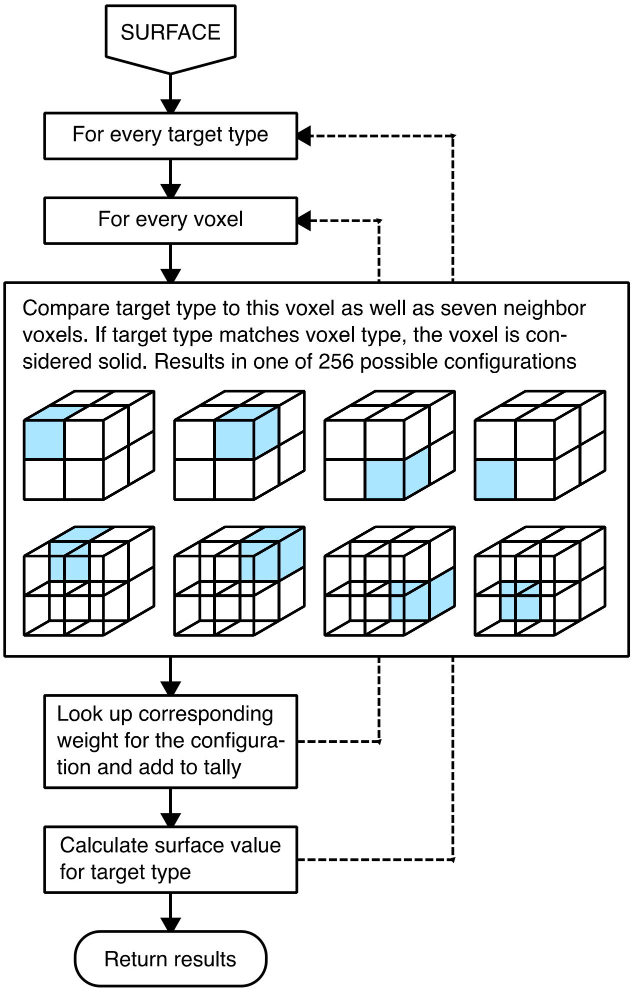

Figure S3. Flow chart depicting the algorithm that is used to calculate surface areas in MoloVol. Here, eight voxels are effectively evaluated at once. Each voxel is considered "solid" when it matches the voxel type of interest. Each of the 256 possible configurations have a corresponding weight. To calculate the surface, these values are summed and multiplied by the surface unit. This algorithm is described in section 4.4.2 of the manuscript. The off-page connector labelled "SURFACE" connects to Fig. 5 of the manuscript.

2. Analytical volume and surface area values for acetylene

The acetylene structure analyzed herein is a linear arrangement of four hard spheres. The V_{vdw}, V_{acc}, S_{vdw}, and S_{acc} values can be readily calculated using formulas for volume and surface areas of spheres and spherical caps. V_{mol} and S_{exc} can also be calculated analytically; however, the calculation is slightly more difficult. The geometric solid that needs to be considered for V_{mol} and S_{exc} alternates between atom-defined and probe-defined sections along the axis of binding on which the atoms are arranged. A 2D slice of this solid is shown in Fig. S4. The atom-defined sections can be readily calculated using the formulas for spherical caps.

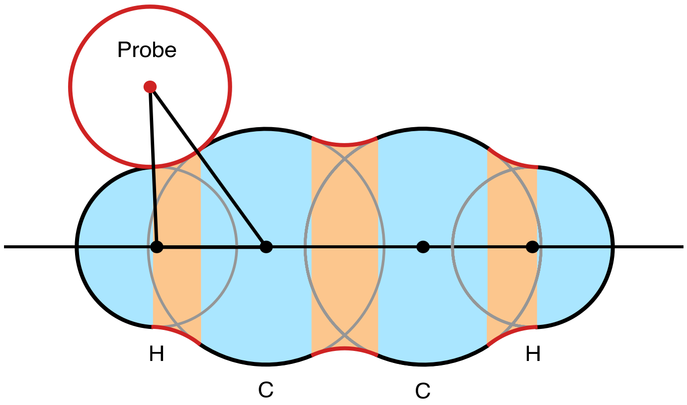

Figure S4. Slice of an acetylene molecule. A rolling probe defines a probe excluded surface and a molecular volume. Surface areas and volumes can be calculated with formulas for spherical caps for blue sections (atom-defined), and with formulas for solids of revolution for orange sections (probe-defined).

To calculate the remaining probe-defined sections, it is necessary to derive a function that gives the radius r of the solid for a position x along the axis of rotation (equivalent to the axis of binding, because the molecule is linear). With this function $r(x)$, the volume V and surface area A of the section can be calculated using formulas for solids of revolution.

$$V = \pi \int_{x_1}^{x_2} r(x)\, dx \tag{1}$$

$$A = 2\pi \int_{x_1}^{x_2} r(x) \sqrt{1 + \left(\frac{d}{dx} r(x)\right)^2}\, dx \tag{2}$$

The radius function can be obtained entirely by using trigonometry on the triangle spanned by the centers of a pair of atoms and a probe (compare Fig. S4). For the following equations the x-axis is parallel to the axis of rotation. The origin is placed where the axis of rotation intersects with the line perpendicular to the axis of rotation passing through the probe center. Therefore, the x-coordinate of the probe is 0. With this reference system the radius function is:

$$r(x) = d_p - \sqrt{r_p^2 - x^2} \tag{3}$$

Here, r_p is the probe radius and d_p is the shortest distance of the probe from the axis of rotation, which depends on the side lengths of the triangle, *i.e.*, the atom-probe distances and the atom-

atom distance. Only the integration limits need to be determined. They are, again, fully determined by the aforementioned triangle and therefore easily calculated:

$$x_1 = r_p \cos(\pi - \alpha) \tag{4}$$

$$x_2 = r_p \cos\beta \tag{5}$$

Here, α and β are the angles of the triangle's vertices centered at atoms A and B respectively. They can be calculated using the law of cosine.

To calculate the volume and surface area, equation (3) is integrated in equations (1) and (2) respectively using the limits from equations (4) and (5). The calculations need to be conducted for the hydrogen-carbon and the carbon-carbon pair.

The geometrical features of the other structures are too complex to easily calculate volumes and surface areas analytically.

3. Pratical aspects of computations

3.1. Hardware specifications

Computations were performed on a 13-inch MacBook Pro from Apple, produced in 2020 (uniquely identified as MacBookPro17,1). The central processing unit (CPU) is an ARM-based Apple M1 and contains an in-built graphics processing unit. The computer provides 8 GB of random-access memory (RAM).

Computation results were verified on a second computer equipped with an Intel Core i7-9750H CPU (2.60 GHz) and 16 GB of RAM.

3.2. Common considerations

Programs were compiled from source code using Apple clang v13.0.0 and gfortran on macOS Big Sur (11.5.2 (20G95)) or MinGW-w64/GCC v10.2.0 on Windows 10 (Version 21H2).

Runtime measurements were conducted using the in-built `time`-command within Apple's bash shell. For the total runtime, the "user" and "sys" values were added. The result represents the total CPU time and is not affected by idling.

For all programs using voxelated space (*i.e.*, MoloVol, 3V, Platon, and PoreBlazer), the *grid resolution* (sometimes called *grid size* or *grid step*) was set to 0.2 Å.

Element radii were set according to MoloVol's default settings:

C 1.77 Å, H 1.20 Å, N 1.66 Å, O 1.50 Å, Cu 2.38 Å, Fe 2.44 Å, Ni 2.40 Å, S 1.89 Å, Zn 2.39 Å.

The *probe radius* was set to 1.20 Å.

Platon, Zeo++ and PoreBlazer can only analyze crystal unit cells. Therefore, isolated molecules such as acetylene were placed in a dummy orthogonal unit cell large enough to let the probe roll freely around the molecule. The total unit cell volume used in some calculations is named $V_{unit\text{-}cell}$.

3.3. MoloVol

MoloVol v1.0.0 or an equivalent development version was used.

A full calculation of MoloVol v1.0.0 was conducted with the following parameters:
Include HETATM: no (when applicable)
Analyze crystal unit cell: yes for HKUST-1, no for other structures
Analyze surface area: yes
Enable two-probe mode: no
Optimization depth: 4

V_{vdw}, V_{mol}, S_{vdw}, S_{exc}, S_{acc}, and S_{occ} are all given directly in the report file.

V_{acc} is calculated as $V_{vdw} + V_{void} + V_{shell}$ (all given in the report file).

3.4. PyMOL

PyMOL v2.4.0 Open-source was used for the calculations.

Element radii were set using the following command (example for carbon)

```
alter (elem C),vdw=1.77
rebuild
```

The following settings were used:

- solvent_radius: 1.2
- dot_density: 4
- dot_solvent: 0 for S_{vdw} and 1 for S_{acc}

Then the command `get_area` was used to calculate S_{vdw} or S_{acc}.

3.5. 3V

3V v1.3 source code was downloaded from https://github.com/vosslab/vossvolvox on 11-02-2022. 3V was compiled as provided by default, *i.e.*, without OpenMP.

Values were obtained with the following commands:

V_{vdw}: `VDW.exe -i input.xyzr -g 0.2`

V_{mol}: `Volume.exe -i input.xyzr -p 1.2 -g 0.2`

V_{acc}: `Volume.exe -i input.xyzr -p 1.2 -g 0.2`

S_{vdw}: `VDW.exe -i input.xyzr -g 0.2`

S_{exc}: `Volume.exe -i input.xyzr -p 1.2 -g 0.2`

S_{acc}: `VDW.exe -i input.xyzr -g 0.2` (with element radii increased by 1.2 Å in the input.xyzr file)

3.6. Platon

Platon was downloaded and compiled on 03-09-2021.

The element radii were set with the command `SET VDWR`.

V_{vdw} was calculated from $V_{unit\text{-}cell} - (V_{void} + V_{core} + V_{shell})$

The total empty space ($V_{void} + V_{core} + V_{shell}$) was obtained with the command
`CALC SOLV GRID 0.2 PROBE 0`

V_{mol} was calculated from $V_{unit\text{-}cell} - V_{occ}$

The probe occupied space V_{occ} was obtained with the command
`CALC SOLV GRID 0.2 PROBE 1.2`

Note that a large error was obtained for V_{vdw}. Thus, it is possible that the program is not designed to process a probe with zero radius.

3.7. Zeo++

Zeo++ v0.3 source code was downloaded from http://www.zeoplusplus.org/zeo++-0.3.tar.gz on 03-02-2022 and compiled following instructions from http://www.zeoplusplus.org/download.html.

Loading custom element radii from a .rad file failed. Thus, we hardcoded the element radii in the source code file networkinfo.cc prior to compilation.

Calculations with Zeo++ must specify a sampling number. For optimal results we used a value of 100000 for all calculations. For optimal runtime, we used 100000 only for volume calculations and 2000 for surface calculations.

Values were obtained with the following commands:

V_{core}: `network.exe -ha -vol 1.2 1.2 100000 output-Vcore.vol input.cif`

V_{occ}: `network.exe -ha -volpo 1.2 1.2 100000 output-Vocc.vol input.cif`

V_{geo} (= V_{void} + V_{core} + V_{shell}):

`network.exe -ha -vol 0 0 100000 output-Vgeo.vol input.cif`

S_{vdw}: `network.exe -ha -sa 0 0 100000 output-Svdw.sa input.cif`

S_{acc}: `network.exe -ha -sa 1.2 1.2 100000 output-Sacc.sa input.cif`

Then the desired values were derived as follow:

V_{vdw}: $V_{unit\text{-}cell} - V_{geo}$

V_{mol}: $V_{unit\text{-}cell} - V_{occ}$

V_{acc}: $V_{unit\text{-}cell} - V_{core}$

3.8. PoreBlazer

PoreBlazer v4.0 was downloaded and compiled from https://github.com/SarkisovGroup/PoreBlazer on 03-03-2022.

Element diameters were set in the file UFF.atoms.

The probe diameter (2.4 Å) and grid resolution were set in the file default.dat as *Nitrogen atom sigma* and *Cubelet size*, respectively. Other parameters from default.dat were kept as provided.

The structures were provided in the XYZ format and the unit cell parameters were set in the file input.dat.

The calculation produced the following values in results.txt:

S_{acc} named *Total surface area*

V_{geo} (= V_{void} + V_{core} + V_{shell}) named *Total geometric volume*

V_{occ} named *Total probe-occupiable volume*

Then the desired values were derived as follow:

V_{vdw}: $V_{unit\text{-}cell} – V_{geo}$

V_{mol}: $V_{unit\text{-}cell} – V_{occ}$

4. Computational results

Volumes and surface areas calculated for five structures including small molecules, an open cage compound, a protein, and a porous material (metal-organic framework) with six different programs are reported in Table S1. The metal-organic framework was not analyzed with PyMOL and 3V because these programs are unsuitable to analyze unit cells of crystal structures.

Table S1. Comparison of volumes and surface areas of molecules calculated with different programs. Values in brackets show the relative error compared to the analytical result.

Structure	Method	V_{vdw} [Å^3]	V_{mol} [Å^3]	V_{acc} [Å^3]	S_{vdw} [Å^2]	S_{exc} [Å^2]	S_{acc} [Å^2]
**Acetylene**	Analytical	37.80	37.95	153.75	57.47	56.55	141.82
	MoloVol	37.74 (–0.16%)	37.84 (–0.29%)	153.74 (–0.007%)	57.00 (–0.82%)	56.98 (0.76%)	141.48 (–0.24%)
	PyMOL	-	-	-	57.52 (0.087%)	-	142.05 (0.16%)
	3V	37.84 (0.11%)	40.08 (5.61%)	153.90 (0.098%)	55.68 (–3.11%)	58.20 (2.92%)	139.50 (–1.64%)
	Platon	23 (–39%)	40 (5.4%)	-	-	-	-
	Zeo++	38.79 (2.62%)	39.00 (2.77%)	153.74 (–0.007%)	57.41 (–0.10%)	-	141.91 (0.063%)
	PoreBlazer	35.80 (–5.29%)	35.90 (5.40%)	-	-	-	165.78 (16.9%)
**Fullerene C_{60}**	MoloVol	528.62	537.66	1077.97	400.10	385.38	526.75
	PyMOL	-	-	-	404.28	-	529.93
	3V	528.90	555.50	1077.08	397.74	385.99	520.99
	Platon	405	555	-	-	-	-
	Zeo++	531.43	566.91	1080.88	404.53	-	527.27
	PoreBlazer	508.47	519.28	-	-	-	536.03
**Norcorrole based Metal-Organic Cage**	MoloVol	5012.63	5713.31	10297.38	4742.73	3487.44	4125.60
	PyMOL	-	-	-	4890.08	-	4184.12
	3V	5012.47	5913.69	10298.94	4695.69	3459.31	4099.09
	Platon	3637	5889	-	-	-	-
	Zeo++	4990.7	6027.1	10306.4	4886.08	-	4177.86
	PoreBlazer	4863.13	5589.22	-	-	-	4181.57
**Trypsin Protein 1C2H**	MoloVol	21114.77	27185.46	38129.16	22177.08	8915.63	9336.11
	PyMOL	-	-	-	22781.00	-	9490.06
	3V	21115.15	27666.52	38129.50	21920.60	8857.17	9297.21
	Platon	14858	27499.7	-	-	-	-
	Zeo++	21350	30080	38477	22943.8	-	9483.98
	PoreBlazer	20813.04	27371.54	-	-	-	9107.67
**Metal-Organic Framework HKUST-1**	MoloVol	5849.64	6128.41	11731.17	4963.34	4561.96	4544.44
	Platon	4416.9	6281.4	-	-	-	-
	Zeo++	5838.67	6466.47	11757.09	5036.32	-	4579.4
	PoreBlazer	5737.69	6024.79	-	-	-	4100.95

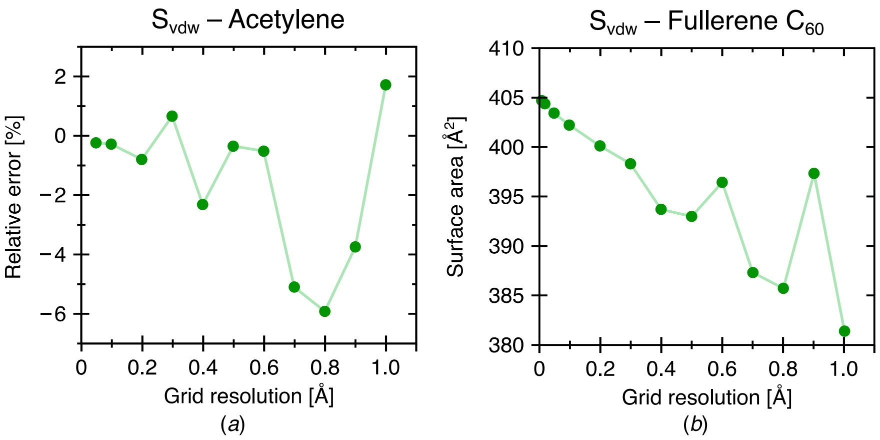

Figure S5. Van der Waals surface area calculated with MoloVol at different resolutions for acetylene (a) and fullerene C_{60} (b). Connecting lines have been added for visual clarity.

5. Breakdown of runtime contributions

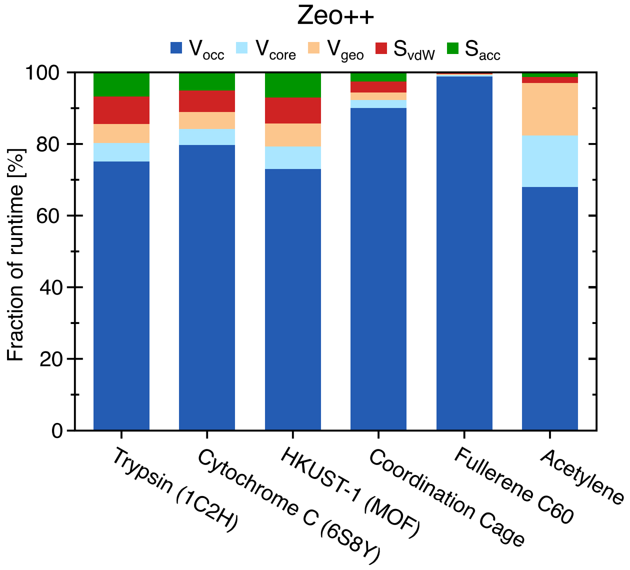

Figure S. Breakdown of runtime contributions for Zeo++ calculations. Each volume and surface area value is calculated in a separate calculation.

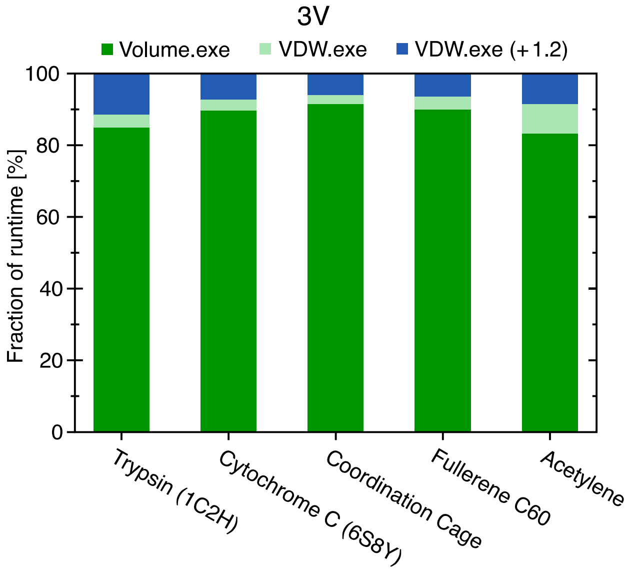

Figure S7. Breakdown of runtime contributions for 3V calculations. To calculate S_{acc}, the atomic radii were increased by the probe radius (1.2 Å), then S_{vdw} was computed using the VDW executable. The results shown here were obtained without OpenMP.

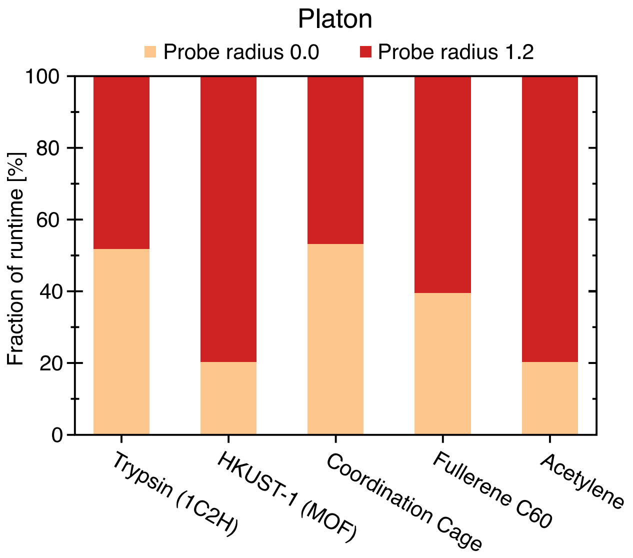

Figure S8. Breakdown of runtime contributions for Platon calculations. To calculate V_{vdw}, the volume calculation was repeated with a probe radius of 0. However, it appears that this workaround does not produce reliable results (see Table S1).

6. Input structures

6.1. Acetylene

A model of acetylene was used with atomic coordinates given in Table S2. When a unit cell was necessary, the acetylene molecule was placed in a dummy unit cell of size 10×10×10 Å^3.

Table S2. Cartesian coordinates of acetylene in Å.

H	2.160000	3.848000	3.150000
H	5.097000	2.152000	3.150000
C	3.104000	3.303000	3.150000
C	4.153000	2.697000	3.150000

6.2. Fullerene C_{60}

A model of fullerene C_{60} was used with atomic coordinates given in Table S3. When a unit cell was necessary, the C_{60} molecule was placed in a dummy unit cell of size 13×13×13 Å^3.

Table S3. Cartesian coordinates of fullerene C_{60} in Å.

C	4.931317	8.254983	8.921017
C	3.740317	7.909983	8.151017
C	3.758317	8.691983	6.919017
C	3.232317	4.616983	6.869017
C	2.815317	6.014983	6.886017
C	3.282317	6.607983	8.135017
C	5.684317	9.250983	8.166017
C	4.959317	9.520983	6.929017
C	5.598317	7.279983	9.634017
C	5.113317	5.903983	9.617017
C	6.272317	5.017983	9.605017
C	4.051317	8.364983	4.500017
C	3.316317	8.130983	5.739017
C	2.832317	6.753983	5.722017
C	3.957317	4.345983	8.106017
C	3.988317	5.576983	8.888017
C	7.056317	7.243983	9.632017
C	7.473317	5.845983	9.614017
C	4.020317	7.133983	3.717017
C	3.266317	6.138983	4.472017
C	5.186317	9.148983	4.509017
C	5.653317	9.742983	5.757017
C	7.112317	9.706983	5.755017
C	7.413317	3.450983	8.091017
C	6.243317	3.852983	8.865017
C	5.053317	3.507983	8.094017
C	8.549317	4.234983	8.100017
C	9.283317	4.468983	6.861017
C	9.768317	5.845983	6.878017
C	8.956317	8.565983	6.913017
C	8.939317	7.783983	8.145017
C	7.769317	8.185983	8.918017
C	3.643317	4.033983	5.687017
C	4.802317	3.147983	5.675017
C	5.536317	3.382983	4.436017
C	8.580317	5.465983	8.883017
C	9.333317	6.460983	8.127017
C	6.946317	2.856983	6.843017
C	5.488317	2.892983	6.845017
C	6.356317	8.746983	3.735017
C	7.546317	9.091983	4.506017

C	3.661317	4.815983	4.455017
C	4.831317	4.413983	3.682017
C	7.797317	9.451983	6.925017
C	7.063317	9.216983	8.164017
C	9.367317	7.983983	5.731017
C	8.643317	8.253983	4.494017
C	8.612317	7.022983	3.712017
C	7.002317	5.319983	2.966017
C	5.543317	5.355983	2.968017
C	5.126317	6.753983	2.986017
C	7.669317	4.344983	3.679017
C	8.859317	4.689983	4.449017
C	8.841317	3.907983	5.681017
C	9.784317	6.584983	5.714017
C	9.317317	5.991983	4.465017
C	6.916317	3.348983	4.434017
C	7.640317	3.078983	5.671017
C	7.486317	6.695983	2.983017
C	6.327317	7.581983	2.995017

6.3. Metal-organic cage

The crystal structure of a previously reported tetrahedral Fe_4L_6 metal-organic cage with an open cavity was used (Yamashina, M., Tanaka, Y., Lavendomme, R., Ronson, T. K., Pittelkow, M. & Nitschke, J. R. (2019). *Nature* **574**, 511–515).

The original cif file (CCDC 1893553) was converted to xyz format with the program Mercury 2021.3 to contain a single cage. When a unit cell was necessary, the cage complex was placed in a dummy unit cell of size 30×30×30 $Å^3$.

6.4. Cytochrome C complex

The crystal structure of the cytochrome C complex was downloaded from the protein data bank (PDB 6S8Y) in the pdb format. HETATM were included for calculations. The file was converted to xyz format with the program Mercury 2021.3. When a unit cell was needed, the protein was placed in a dummy unit cell of size 38×68×45 Å3.

6.5. Trypsin protein

The crystal structure of trypsin was downloaded from the protein data bank (PDB 1C2H) in the pdb format. HETATM lines were removed, and the file was converted to xyz format with the program Mercury 2021.3. When a unit cell was needed, the protein was placed in a dummy unit cell of size 60×50×60 $Å^3$.

6.6. HKUST-1

The crystal structure of this porous metal-organic framework can be accessed from the CCDC (number 755080). The same structure is provided as an example with PoreBlazer on GitHub repository: https://github.com/SarkisovGroup/PoreBlazer/tree/main/Windows/HKUST1
